# Species composition, relative abundance, and habitat association of birds in Dodola dry evergreen afro-montane forest and sub-afro-alpine scrubland vegetation, southeast Ethiopia

**DOI:** 10.7717/peerj.16775

**Published:** 2024-01-11

**Authors:** Zenebe Ageru Yilma, Girma Mengesha, Zerihun Girma

**Affiliations:** 1 Natural Resource, Mizan ATVET College, Mizan Teferi, SWERS, Ethiopia; 2 School of Wildlife and Eco-tourism, Hawassa University, Hawassa, Ethiopia; 3 Wondo Genet College of Forestry and Natural Resource, Hawassa University, Wondo Gent, Ethiopia

**Keywords:** Distance Sampling, Birds, Relative abundance, Diversity, Species, Dodola

## Abstract

**Background:**

Birds’ functional groups are useful for maintaining fundamental ecological processes, ecosystem services, and economic benefits. Negative consequences of loss of functional groups are substantial. Birds are usually found at a high trophic level in food webs and are relatively sensitive to environmental change.

**Methods:**

The first surveillance bird study was carried out southeast of Ethiopia adjacent to Bale Mountain National Park aimed at investigating the composition, relative abundance, and distribution of Aves. Using regular systematic point transact sampling, the density and species composition were analyzed through the mark recapture distance sampling engine assisted by R statistical software.

**Results:**

This study recorded a total of seventy-eight bird species over two distinct seasons. Among these, fifteen species were exclusive to Erica habitats, twenty-six were found in natural forest habitats, and three were specific to plantation forest habitats. The study also discovered three endemic species. Based on the 2018 IUCN Red List categories, six of the species are globally threatened, three are near threatened, and the remaining sixty-nine are classified as least concern. The relative abundance of birds did not significantly differ across habitats and seasons, but variations were observed among blocks. Bird density was found to fluctuate across the three habitats and two seasons; however, these habitat differences were not influenced by seasonal changes.

**Conclusion:**

The findings of this study reveal that the differences in composition and relative abundance are not merely seasonal changes in the forest and Erica habitats. Instead, these habitats create microclimates that cater to specific bird species. However, this localized endemism also presents challenges. The concentration of endemic species and potential resource constraints could pose a threat to these habitat-specialist birds.

## Introduction

Birds, which encompass a remarkable diversity of over 11,000 species, are a captivating and highly valued part of the natural world (BirdLife International, 2018). Their intricate variety ranges from the tiniest to the largest, and the slowest to the swiftest flyers. Each bird species possesses a unique presence, habits, and habitat preferences (BirdLife International, 2018). This remarkable diversity showcases itself in both the vast numbers of some species, like the 8,421 species classified as least concern, and the scarcity of others, with a mere handful of surviving individuals (IUCN, 2018). The International Union for Conservation of Nature (IUCN) red list categories further categorize birds, with 1,470 species classified as threatened, and among them, 223 critically endangered, 461 endangered, and 786 vulnerable (IUCN, 2018)

In this tapestry of avian diversity, Ethiopia emerges as a hotspot, harboring 872 distinct bird species, 18 of which are endemic, and another 67 represented as endemic sub-species (Mengistu, 2002). With 851 of its bird species evaluated within the IUCN red list categories, Ethiopia underscores the global importance of preserving avian populations (IUCN, 2018). As they traverse the world’s diverse habitats, birds leave their ecological footprints, indicating the health of ecosystems. Birds, being excellent indicators of environmental health, offer a window into the impacts of pollution and climate change (Sekercioglu, Daily & Ehrlich, 2004).

The interplay between birds and their habitats is fundamental in shaping distribution patterns. Habitats, often shaped by vegetation and complemented by other factors, determine where birds thrive. Recognizing the significance of this dynamic, Important Bird and Biodiversity Areas (IBAs) and Key Biodiversity Areas (KBAs) have emerged as key tools for global conservation efforts. These designated areas, which number over 13,000 across more than 200 countries, act as crucial bastions for the conservation of biodiversity (BirdLife International, 2018).

Beyond their ecological roles, birds provide an array of essential ecosystem services. They diligently contribute to pollination, insect pest control, seed dispersal, and nutrient cycling, all which ripple through ecosystems, benefiting both nature and human society. Bird activity knits together ecosystems and influences the abundance of other species (Sekercioglu, Daily & Ehrlich, 2004; Wenny et al., 2011). For example, frugivorous birds maintain gene flow and enhance restoration efforts through seed dispersal. In this context, birds can be regarded as ecological engineers, shaping landscapes, and fostering ecosystem resilience (Wenny et al., 2011).

However, the intricate web of avian diversity and its contributions to ecosystems faces a looming threat. Birds have become bioindicators of environmental changes, and their declining populations serve as a stark warning (Bonisoli-Alquati et al., 2022; Mekonen, 2017). The IUCN red list data reveals a steady deterioration in the status of the world’s bird species (IUCN, 2018). Human activities, from agricultural expansion and logging to pollution and invasive species introduction, are driving these declines (Malhotra, 2022). Furthermore, the long-term specter of climate change hovers, potentially amplifying these threats (de Moraes et al., 2020).

The decline in avian diversity worldwide due to human activities and climate change poses a threat to the ecosystem services that birds provide. Therefore, there is an urgent need for conservation efforts to preserve avian diversity and safeguard these ecosystem services for the benefit of both nature and humanity. The objective of this study was to identify species diversity and relative abundance as baseline information through a survey or census of bird populations in Dodola forest. Initial surveillance or inventory of bird species has not been specifically conducted in the study area. The area is experiencing habitat disturbance, and the status of bird populations remains largely unknown, making this a critical concern. Therefore, it is essential to assess the composition, abundance, and presence or absence of birds across different habitats. This information is crucial for ongoing monitoring and evaluation of bird statuses in the study area. This baseline information would be used to inform conservation efforts and monitor changes in bird populations over time.

## Materials and Methods

### Description of the study area

#### Location

**Figure 1:**
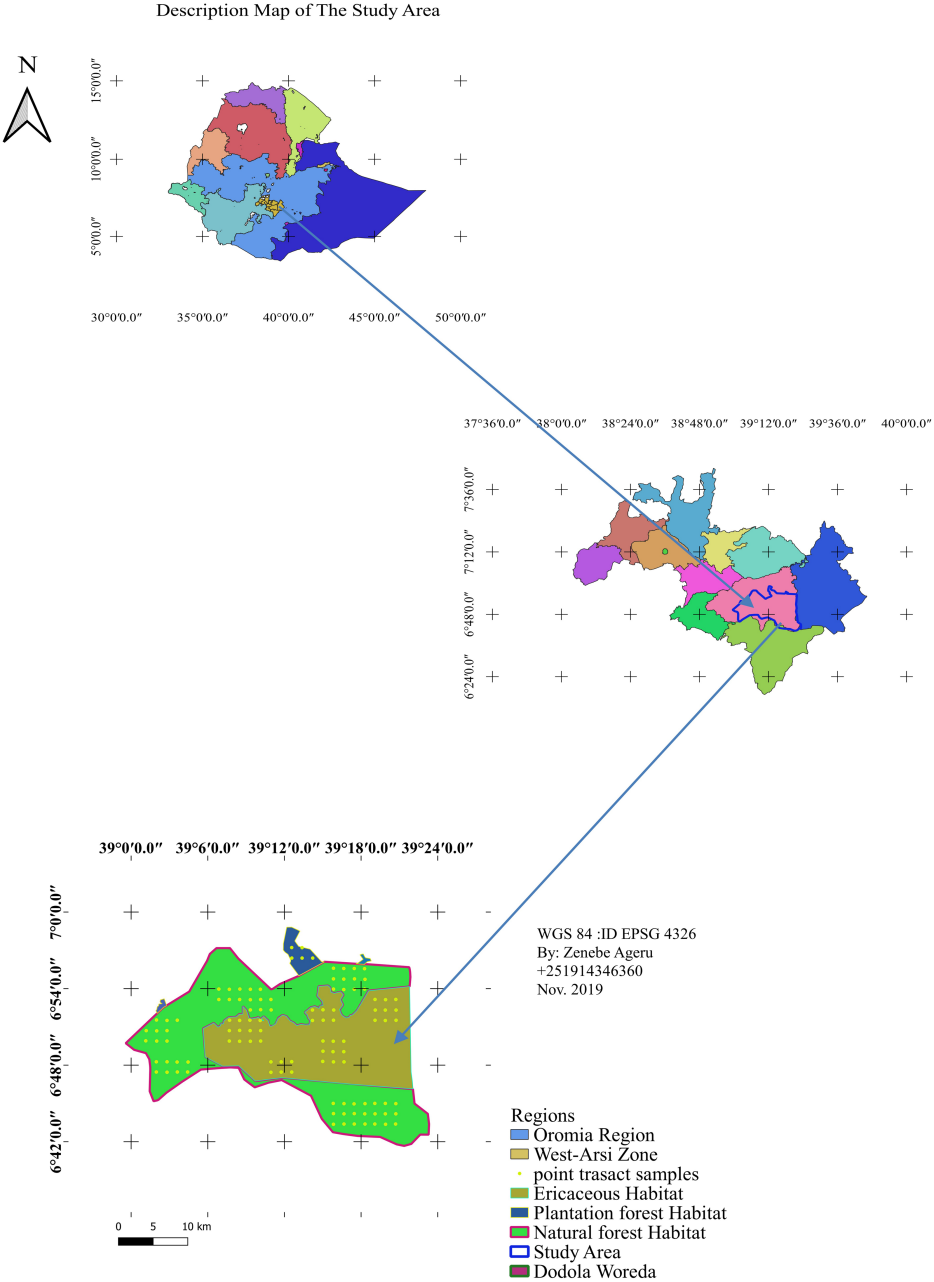
Location map of the study area. Map credit: Zenebe Ageru Yilma.

The Dodola natural forest habitat is part of the Adaba Dodola Jalo forest which is one of the 61 National Forest Priority Areas (NFPA) of the country that covers approximately 530 km^2^ (Gelashe, 2017). The Ericaceous sub-afro alpine habitat is found at higher elevations to the natural forest, while the plantation forest below the dry evergreen afro-montane forest. Dodola forest is located West Arsi zone of the Oromia regional state, southeastern Ethiopia (Fig. 1). The study area is adjacent the Bale mountains massif and occurs at 325 km from Addis Ababa towards the southeast, 70 km from Shashemene. The area is bordered by the Kofale district to the west, the Adaba district to the east, the Nensabo and Kokossa districts to the south, and the Asasa district to the north. The geographical location ranges between 6°39′E38°57′N and 7°0′E39°24′N. The altitude range varies from 2,400–3,712 m.a.s.l. The area is a part of tropical forest and tropical shrub land that consists of natural forest (Dry evergreen Afromontane Forest), Ericaceous vegetation (sub-afro alpine habitat) and community plantation forest of a total of 738.30.24 km^2^.

#### Climate and vegetation

The study area has a four-month as dry season (November–February) and an eight-month as wet season (March–October) (Hundera, Bekele & Kelbessa, 2007). The characteristics of the forest are categorized as upland dry evergreen forests of Afromontane forests (Friis, Rasmussen & Vollesen, 1982). The Dodola region’s forest landscape changes with altitude. Between 2,565 to 2,800 m, conifer forests become dominant, with Podocarpus and Juniperus as the prevailing species. Moving to the middle altitude zone of 2,804–3,115 m, Juniperus procera takes the lead, alongside other broadleaf hardwood species, while Podocarpus falcatus becomes less common and sporadically found at the lower boundary of this zone. In the upper elevation range of 3,120–3,400 m (Brooks, 2009), the forest is similar in ecological characteristics to Bale Mountain National Park, featuring highland forest habitat and sub-afro alpine terrain with Ericaceous vegetation (Evangelista, Swartzinski & Waltermire, 2007). The Erica trimera dominates at higher elevations, while Erica arborea prevails at lower elevations. Additionally, the Dodola region’s forest includes native species like Hagenia abyssinica, *Hypericum lanceolatum*, and Erica arborea, as well as introduced exotic species like Eucalyptus and Cupressus lusitanica in peripheral areas. Juniperus procera is noteworthy for its susceptibility to wildfires and preference for well-drained, nearly neutral pH soils, thriving within specific altitude, precipitation, and temperature conditions in the study area (Gelashe, 2017).

#### Socioeconomic information

The total population of the district is about 194,000. The urban population of 35,000 (18%) is one of the largest in the zone (Ethiopian Central Statistical Agency, 2007). Subsistence agriculture and animal husbandry are the main activities in and outside of the forest delineation area.

### Methods

### Preliminary survey

Preliminary assessment was carried out for identification of key habitats during September 2018. To observe habitat type, age effect, topography, and climatic factors for survey design preconditions. During this period, waypoints were collected using GPS in each habitat type (QGIS.org, 2018). A pilot survey was also conducted for sample size information.

### Sampling design

**Figure 2:**
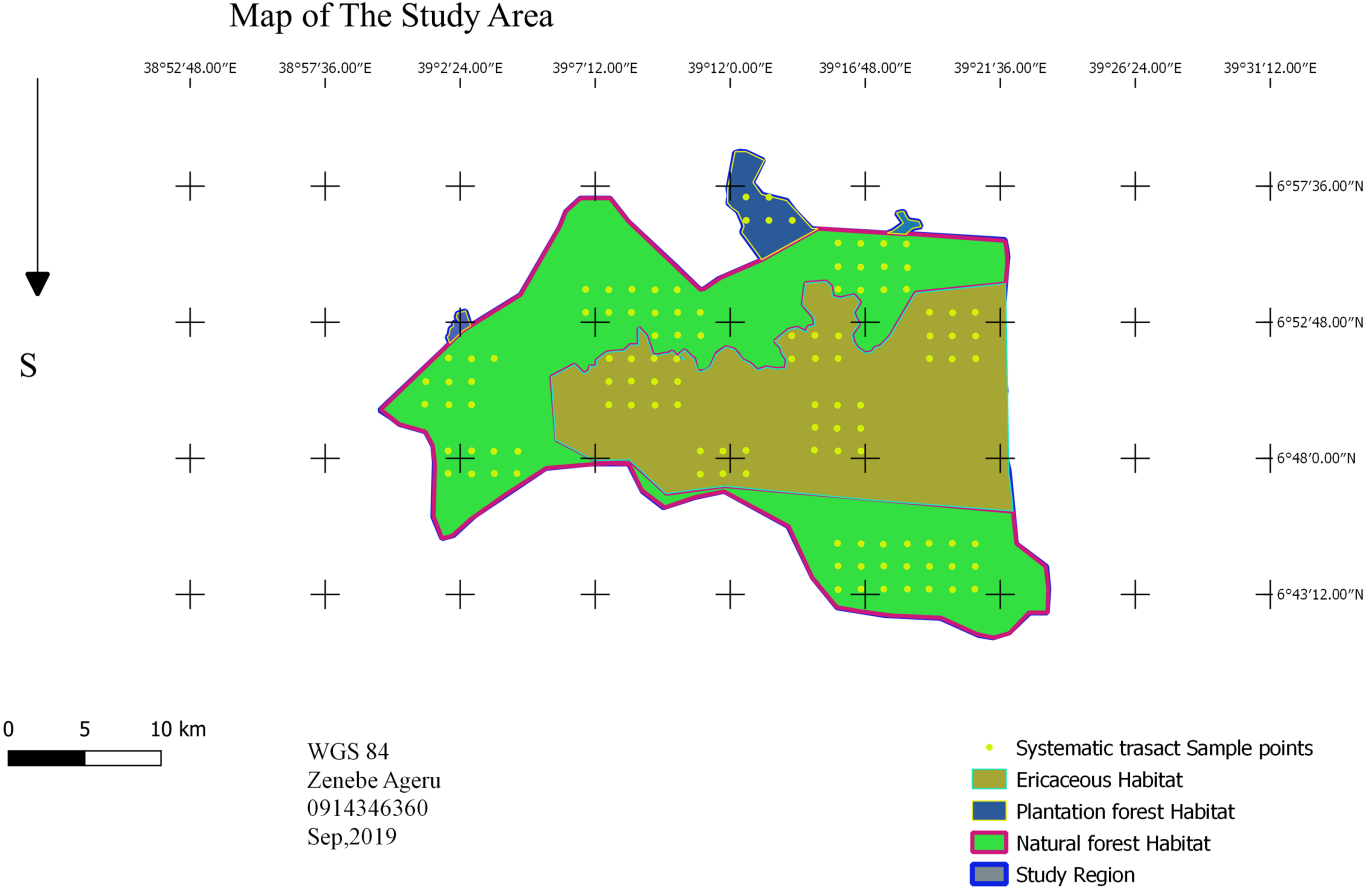
Sampling design. Map credit: Zenebe Ageru Yilma.

A point transect sampling method was used to investigate bird species composition, relative abundance, and habitat association (Buckland et al., 1993). Based on the preliminary survey, the study area was stratified into three dominant habitat types: the sub-afro alpine Ericaceous scrubland habitat; dry evergreen Afromontane Forest; and mixed plantation forest using QGIS. In each habitat type, systematic sampling design was employed. There are eleven blocks: five Erica, five forest and one plantation. The total block area was 128.839 km^2^ area, which is 17.5% of the study area. A systematic point grid of a 1.5-kilometer fixed dimension was randomly superimposed (Fig. 2), and rotation onto the survey region employed proportionally in each habitat type (Buckland et al., 1993). The required number of sample points in the survey region calculated as

(1) \begin{eqnarray*} \frac{b}{(cv(D))^{2}} \ast \frac{k0}{n0} \end{eqnarray*}

where k_0_ and n_0_ are roughly estimated in a pilot survey, and the value of *b* = 3 (Buckland et al., 1993). In the pilot survey there were five points and 54 individual bird observations. The required number of points was 111 points; 42 points in Erica, 64 in forest and five in plantation (Fig. 2). In cluster:

(2) \begin{eqnarray*}k= \frac{k0\{ b+[sd(s)/s]^{2}\} }{nocv{t}^{2}} .\end{eqnarray*}

### Data collection

Field guidebooks were tools for identification of the type of bird species exist in the area (birds of the horn of Africa, birds of the East of Africa, birds of Lake Tana, and important bird areas of Ethiopia) (Redman, Stevenson & Fanshawe, 2016). The data collected was carried out for two seasons during the months of July and August for the wet season and December and January for the dry season. Per season, data collection was conducted in two sessions/visits. Data collection was carried out early in the afternoon and late in the afternoon. Detection distances was measured from the point to detected object (Buckland et al., 1993). All observation beyond 70 m sighting distance were truncated. Birds’ songs were used for most elusive forest birds (Buckland et al., 2001). Identification and counting of most bird species were assisted by binoculars. Points taken 200 m distance inside from edge to avoid edge effect. Duration a point count lasts from 2 min to 20 min (Bibby, Jones & Marsden, 1998).

### Data analysis

Lists of information about habitat type, season, visit, block, point, cluster size and species code were organized in a single data frame. With the help of R software data organizing functions, for similarity and diversity analysis, the data was organized in form of data frame where rows as species list, and columns as the presence and absence data. One column for a single habitat, and one column for a sample point to similarity and species accumulative curve data analysis respectively.

Data analyzed based on distance sampling method distance 7.3 software (Thomas et al., 2010), and the mark recapture distance sampling (MRDS) analysis engine supplemented by R software (R Core Team, 2019). R software was used to analyze ANOVA test using the Car package, and similarity and diversity indicies were analyzed with the Simba and Vegan package (R Core Team, 2019). AIC and the chi-square statistical test were applied to obtain the best-fitted models (Buckland et al., 2001; Buckland et al., 1993). The result was analyzed based on the data recorded on 111 sample points and 222 total efforts of two replication or visit during both seasons. The analysis of distance was based on the formula described by Eqs. (1)–(4) (Buckland et al., 2001; Buckland et al., 1993).

For point transects analyses MRDS always uses the P3 estimator for encounter rate variance.

(3) \begin{eqnarray*}v{a}^{\wedge }{r}_{p3} \frac{n}{T} = \frac{1}{T(k-1)} =\sum _{i=1}^{k}ti( \frac{ni}{ti} - \frac{n}{T} )\end{eqnarray*}

where *ti* is the number of times point *i* was visited, $T={\mathop{\sum }\nolimits }_{I=1}^{K}ti,ni={\mathop{\sum }\nolimits }_{j=1}^{t}nij$ andis $n={\mathop{\sum }\nolimits }_{i=1}^{k}ninj$ the number of objects detected at point *i* on visit *j*.

Relative abundance of avian species determined using encounter rates calculated for each species by dividing the number of birds recorded(n) by the number points (k) multiply time of visit or effort (t) (Buckland et al., 2001).

The encounter rate (ER) was estimated as:

(4) \begin{eqnarray*}ER= \frac{n}{kt} OR \frac{n}{K} .\end{eqnarray*}

Encounter rate data was classified into crude ordinal categories of abundance (*e.g.*, abundant, common, frequent, uncommon, and rare) (Table 1).

**Table 1:** Encounter rates to provide a crude ordinal scale of abundance (Bibby, Jones & Marsden, 1998).

**Abundance category (Number of individuals per 100 field hours)**	**Abundance score**	**Ordinal scale**
<0.1	1	Rare
0.1–2.0	2	Uncommon
2.1–10.0	3	Frequent
10.1–40.0	4	Common
40.0+	5	Abundant

The number of individuals per total effort were ≤0.01, 0.01–0.2, 0.2–1,1-4 and >4. For each interval, the following abundance labels is given rare, uncommon, frequent, common, and abundant, respectively. Therefore, the relative abundance of each bird species was determined by Excel if function of rare, uncommon, frequent, common, and abundant. For example, if the encounter rate is ≤0.01, the species is considered as rare. Analysis were prepared for two type of data selection steps in multispecies analysis options. the first is setting individual species analysis using data filter, the second was not based on individual species; thus, birds as one taxonomic categories of class of Aves as compared to species taxa. In both steps, habitats were stratum whereas seasons were analyzed by using data filter separately.

A two-way ANOVA was used to analyze density and number of individual observation effect of three factors through season, habitat, and species. The ANOVA type III error to investigate interaction effect (Model 1). The ANOVA type II error was used for incasing of non-interaction effect (Model 2)

(Model 1) \begin{eqnarray*}{\mathrm{\mu }}_{ijk}=\mathrm{\mu }+{\alpha }_{i}+{\beta }_{j}+{\gamma }_{k}+{\delta }_{ij}\ldots \ldots \ldots \ldots .\end{eqnarray*}

(Model 2) \begin{eqnarray*}{\mathrm{\mu }}_{ijk}=\mathrm{\mu }+{\alpha }_{i}+{\beta }_{j}+{\gamma }_{k}\ldots \ldots \ldots \ldots .\end{eqnarray*}

where, μ = the overall mean of species observed, *α*_i_, *β*_j_ and *γ*_j_ are the i^th^, j^th^ and k^th^ habitat, season and species effects, respectively. where *δ*_*ij*_ is interaction term (Searle, Speed & Milliken, 1980). *Post-hoc* test used for separate group analysis for interaction effect results. Estimated marginal means (emmeans) was used for non-interaction effect pairwise comparison of groups. Differences were considered statistically significant at 5% (Chambers & Hastie, 1992). Unbiased sim was calculated as $\tau ={\mathop{\sum }\nolimits }_{i=1}^{S}( \frac{{n}_{i}({n}_{i}-1)}{{N}_{i}({N}_{i}-1)} )$ , Simpson’s index D = $\sum \left( { \frac{ni}{N} }^{2} \right) $ Simpson’s Simpson returns *1-D* and inv Simpson returns *1/D* (Hurlbert, 1971) $H=-\sum ( \frac{ni}{N} )\log \frac{ni}{N} $, $E= \frac{H}{\log S} $ where *n*_*i*_ denotes number of individuals in the i ^th^ species (*n*_*i*_ = 1,2,3…., *n* and *n*1 + *n*2…*n* = *N*), S = total number of species (Shannon, 2001). In Fisher’s logarithmic series the expected number of species f with n observed individuals is ${f}_{n}=\alpha \frac{{x}^{n}}{n} $ The parameter *α* is used as a diversity index. The parameter *x* is taken as a nuisance parameter which is not estimated separately but taken to be n/(n+ *α*) (Fisher, Corbet & Williams, 1943). The species discovery curve was used species richness/number of species discovered across each sample points based on the sample-based rarefaction formula for adequate sample size for a multi-species survey.

\begin{eqnarray*}\bar {S}={S}_{n}-{ \left( {n-nk\atop i} \right) }^{-1}\sum _{k\in G} \left( {n-nk\atop i} \right) ,i=1,\ldots ,n \end{eqnarray*}

A collection on *n* samples, the rarefaction curve is the plot of $\bar {Si}$ against *i* (*i* = 1, …, *n*), where *S*_*i*_ indicates the arithmetic mean, *S*_*n*_ denotes the total number of observed species, *nk* denotes the number of samples containing at least one individual species *k* ∈ *G* (Chiarucci et al., 2008).

The diversity and relative abundance presented by tables, qq plot and detection function plot. Statistical difference presented through ggplot2 supported by narrative descriptions. Habitat association of number of species were computed for Sorenson’s similarity index (SI) among habitats under two seasons by using the following formula. SI = 2a/2a+b+c; where 2a = number of species common to two habitats, b = number of species in first habitat, c = number of species in the second habitat (Sorensen, 1948).

## Results

### Species composition

Over the course of two distinct climatic periods (dry and wet), a total of 78 species of birds were recorded. Within the recorded species, the Abyssinian Catbird (*Parophasma galinieri*), Ethiopian Siskin (*Serinus nigriceps*), and Yellow Fronted Parrot (*Poicephalus flavifrons*) have been identified as endemic. Furthermore, there exists a subset of ten species, inclusive of the Wattled Ibis *(Bostrychia carunculate*), the black-winged lovebird (*Agapornis taranta*) and Rouget’s Rail (*Rouget‘s rougetii*), which are recognized as endemic to both Ethiopia and Eritrea (Appendix S1). Based on the lowest AIC value of MRDS analysis engine, the fitted model was single observer distance model and half-normal key function with model for scale parameters is a constant (CDS).

**Figure 3:**
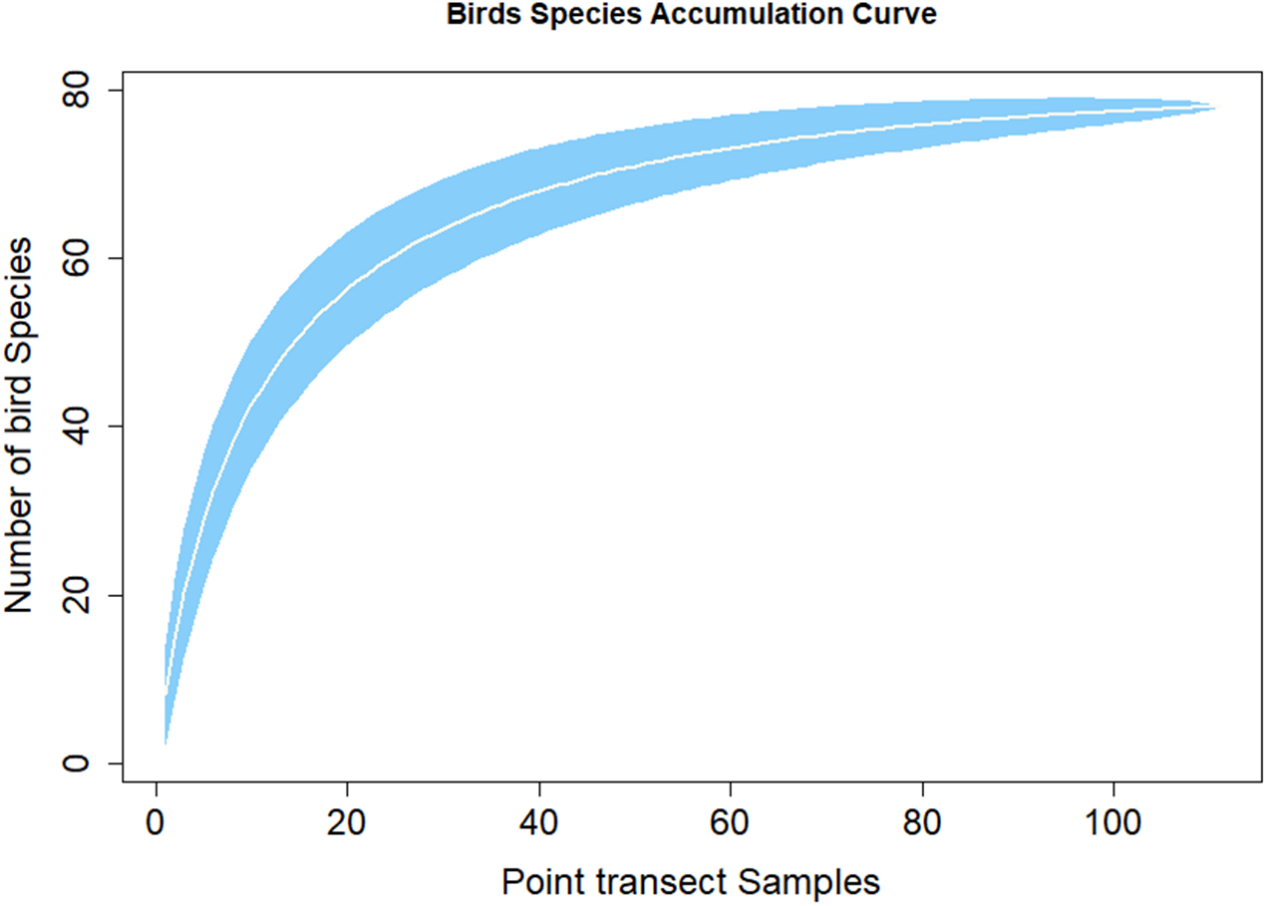
Species accumulation curve.

**Figure 4:**
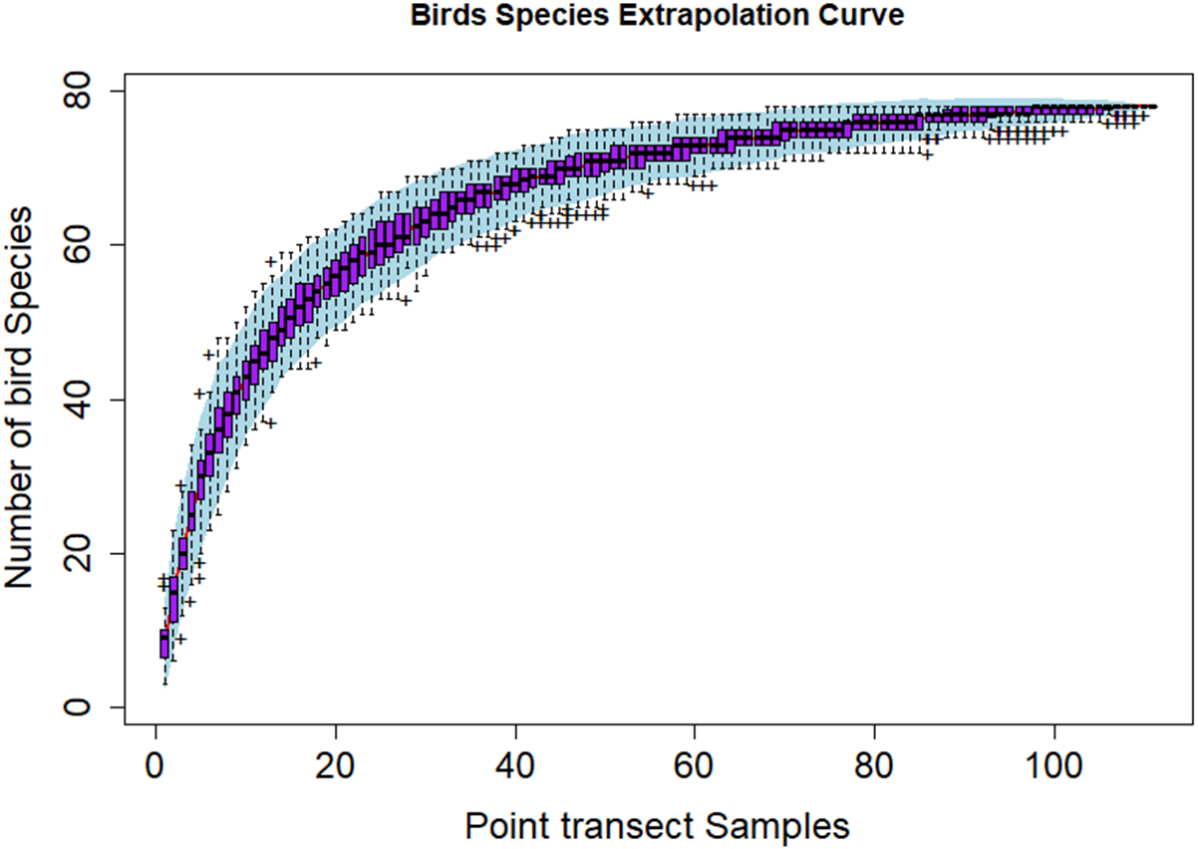
Species extrapolation curve.

Figure 3 shows the species discovery curve and Fig. 4 shows the extrapolation curve with increasing number of species in the *y* axis with sample points in the *x* axis; the curve turns as asymptote shape indicates that the species discover is adequate. The asymptote predicts 86 species to be discovered, which means that over 90% of the species in the area were discovered with a slope 2.62 (the more the slope close to zero, a few or none of species in the area are left detected) (Fig. 3).

**Figure 5:**
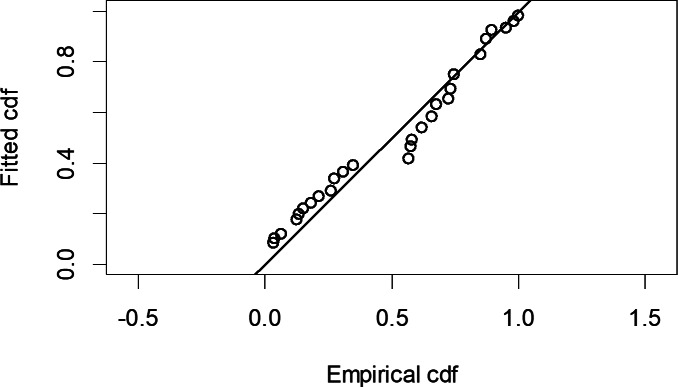
Detection function QQ plot during wet season.

**Figure 6:**
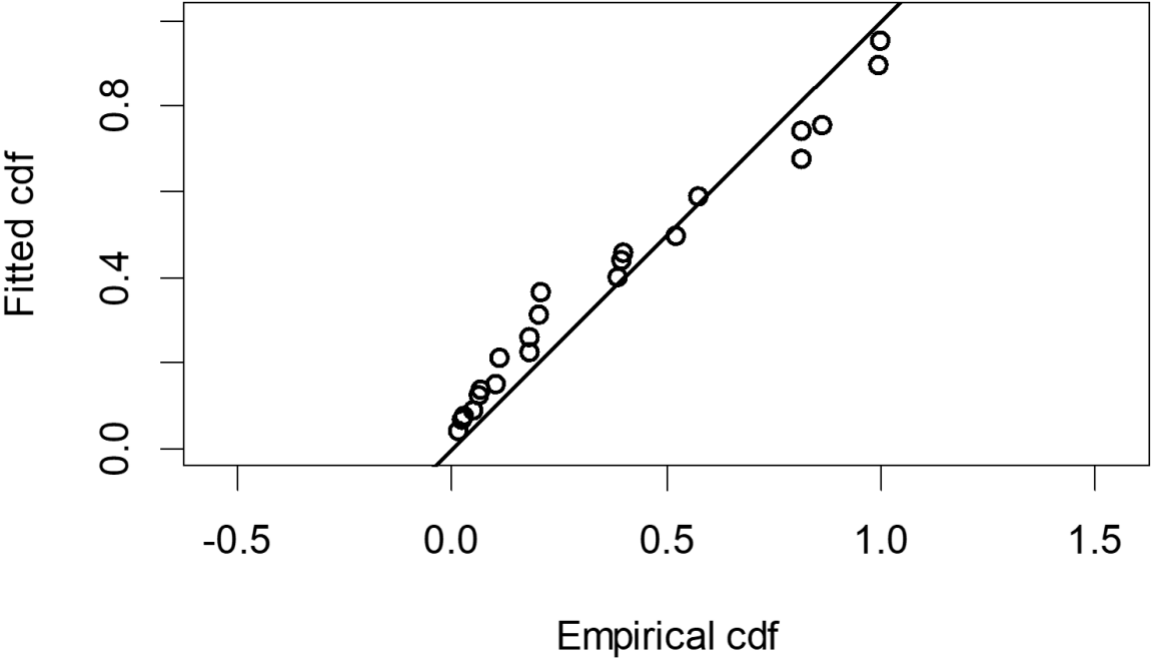
Detection function QQ plot during dry season.

**Figure 7:**
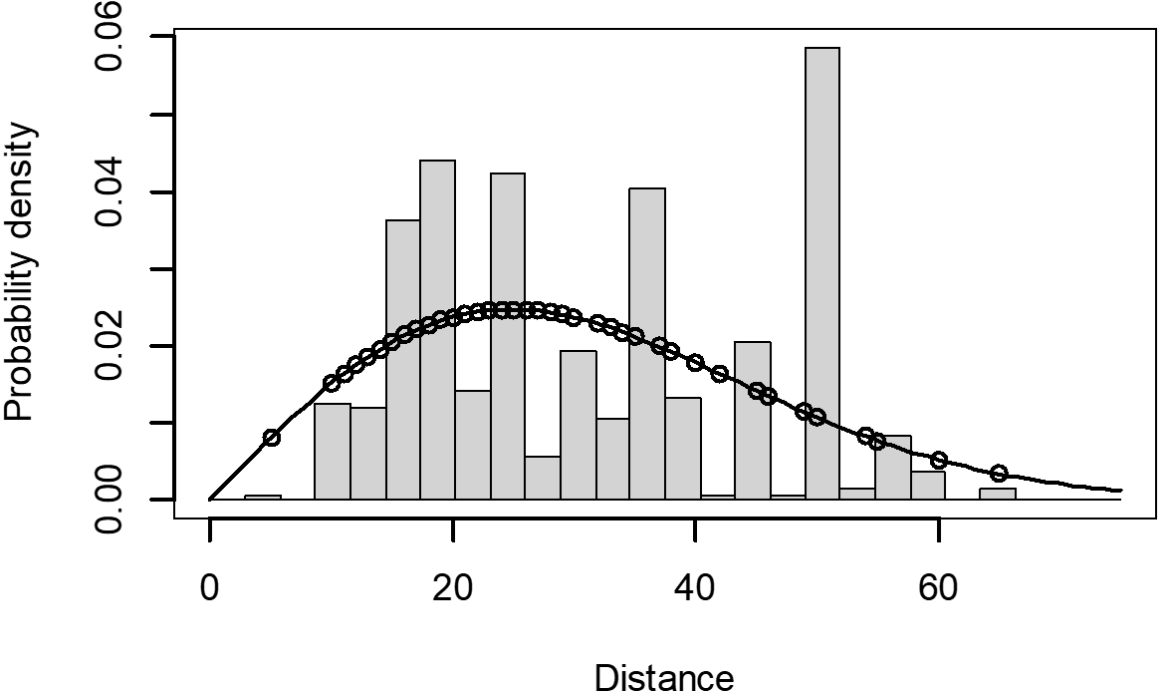
Detection function/plot: detection probability class of birds during wet season. Points indicate probability of detection for a given observation and lines indicate the detection function.

**Figure 8:**
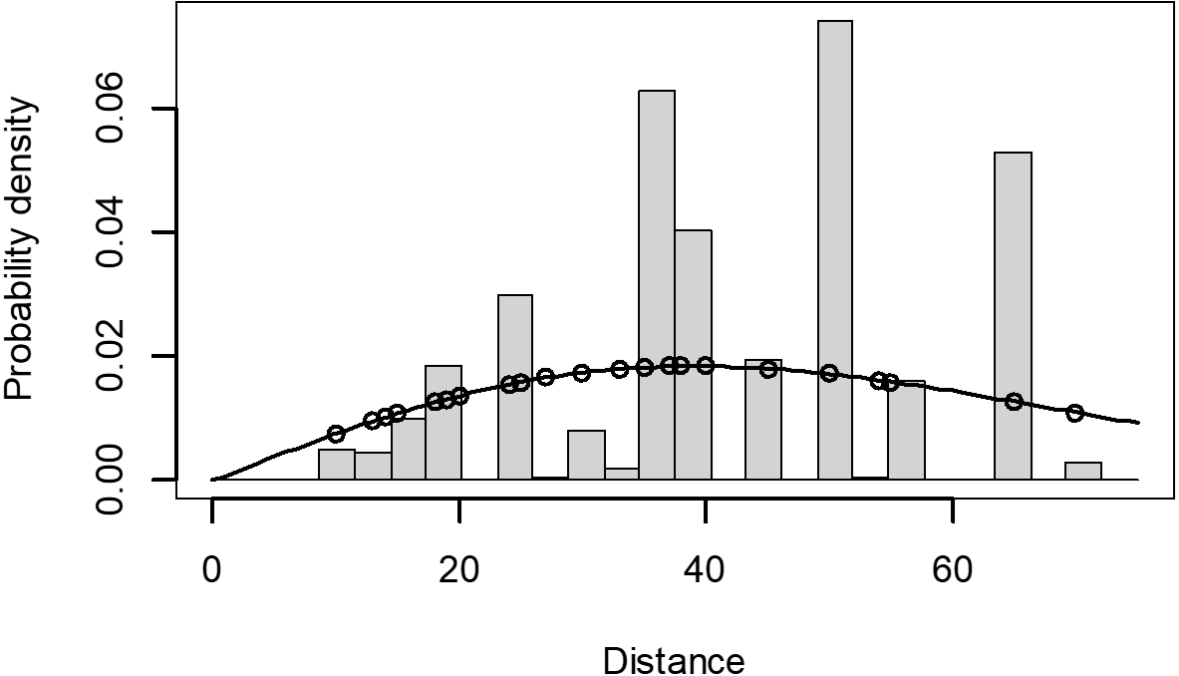
Detection function/plot: detection probability class of birds during dry season. Points indicate probability of detection for a given observation and lines indicate the detection function.

The Quantile-Quantile (QQ) plot, which shows the fitted cumulative distribution function (cdf) against the empirical distribution function (edf), represents the number of observed bird species. The dots on the plot correspond to these observations. The line in the QQ plot represents the expected distribution if the model fit was perfect. The proximity of the dots to the line indicates the fit of the model. In this case, the dots surrounding the line suggest that the model is well-fitted (Figs. 5 and 6). The detection function plots illustrate the expected probability density function of frequencies divided by distance. The curve in these plots represents the expected distribution, while the histograms display the number of observations. The unweighted Cramer-von Mises tests a *p*-value was less than 0.001 in both seasons (Figs. 7 and 8). It is important to note that the detection function depicted in Figs. 6 and 8 represents the overall class Aves. This means it does not account for individual bird species observed in the study.

**Figure 9:**
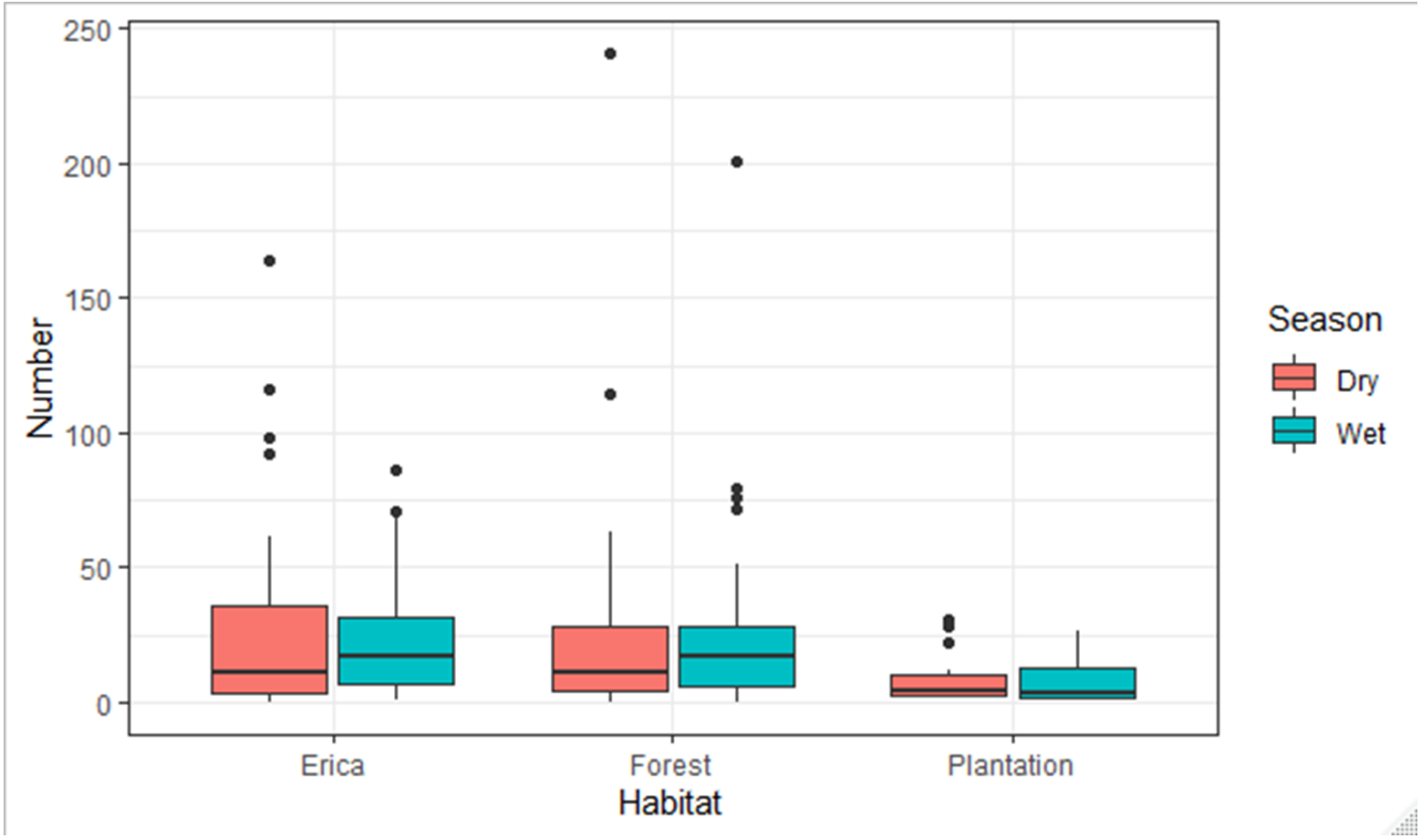
Number of species observed in different habitats during the dry and wet seasons.

The species composition of birds during the wet and dry seasons was not significantly different (F, Season = 0.004, *p* > 0.05) which was 0.95. On the other side, there was a significant difference among habitats (F, Habitat = 12.78, *p* < 0.05) which was 7.466e−06 ***. There was no season and habitat interaction effect (F2, Habitat: Season = 2.28, *p* > 0.05) which was 0.11. The estimated marginal means, also known as least-squares means, revealed a significant difference in the mean number of species across two habitat types: Erica and forest. The mean number of species in the Erica habitat was 24 (±3.16 SE), while in the forest habitat it was 22 (±2.33 SE). However, in the plantation habitat, the estimated marginal mean value was −0.8 (±4.3 SE) (Fig. 9).

According to a Tukey pairwise comparison test with a 95% confidence interval, there was no significant difference in the mean number of species between the Erica and forest habitats. Plantation had the least mean number of species. The *P* value for Erica *vs* forest was >0.05. The *P* value for Erica *vs* plantation and forest *vs* plantation was <0.01.

The highest species diversity (D) during the wet and dry seasons were observed in Forest habitat (dry evergreen afromontane forest), followed by the Erica (sub-afroalpine) habitat with (0.951 & 0.949) and (0.929 & 0.926) respectively, while the mixed plantation habitat had the least with (0.905 & 0.887). The highest species evenness was observed in the Erica habitat. For the entire season, the forest habitat had the highest species diversity (0.943), while Erica habitat had the highest species evenness (0.85) (Table 2).

**Table 2:** Birds species diversity during wet and dry seasons.

Habitat	Season	No. of species	No. of individuals	D	H	Inv	unbiased sim	alpha	H/log(S)
Erica	Dry	36	1,104	0.926	2.94	13.57	0.93	7.13	0.82
	Wet	36	877	0.949	3.20	19.51	0.95	7.57	0.89
	Total	39	1,981	0.941	3.11	17.06	0.94	6.89	0.85
Forest	Dry	58	1,378	0.929	3.28	14.17	0.93	12.26	0.81
	Wet	54	1,366	0.951	3.46	20.44	0.95	11.23	0.87
	Total	59	2,744	0.943	3.41	17.52	0.94	10.61	0.84
Plantation	Dry	18	157	0.887	2.47	8.88	0.89	5.25	0.85
	Wet	22	167	0.905	2.62	10.57	0.91	6.78	0.85
	Total	25	324	0.901	2.63	10.14	0.90	6.32	0.82

### Species relative abundance

In the dry season, a total of 2,639 individual birds were recorded, while in the wet season, 2,410 individual birds of 78 species were observed (Table 3). In the 2018 IUCN red list categories, six species faced global threats, three species neared the threat status, and a total of 69 species were classified as least concern.

**Table 3:** The encounter rate of the three habitats during both seasons number of birds/total effort.

	Dry					Wet				
Region	Effort	N	ER	se.ER	cv.ER	Effort	N	ER	se.ER	cv.ER
Erica	84	1,104	13.14	1.88	0.14	84	877	10.44	1.15	0.11
Forest	128	1,378	10.77	0.91	0.08	128	1,366	10.67	0.93	0.09
Plantation	10	157	15.70	4.38	0.28	10	167	16.70	3.92	0.23
Total	222	2,639	11.89	0.91	0.08	222	2,410	10.86	0.72	0.07

**Notes.**

n total number of observations ER Encounter rate se.ER Standard error for ER cv.ER coefficient variation of ER

The mixed plantation forest habitat recorded the highest relative abundance of Aves, with 15.7 and 16.7 during the dry and wet seasons respectively. This was followed by Erica in the dry season with 13.14, and the forest in the wet season with 10.67. The dry season exhibited a higher overall seasonal relative abundance of 11.89 (Table 3).

The relative abundance of individual species in stratified habitat is shown in Tables 4, 5 and 6 in Erica, forest and plantation habitat, respectively. In the Erica (sub-afroalpine habitat), the encounter rate was calculated as number of individual observations in the Erica per Erica point samples times number of a point visit (n/84). In the Erica habitat, the Red-wing Starling had the highest relative abundance during the dry season (1.95), while the Scare Swift had the highest relative abundance in the wet season (1.02). The Chestnut-napped Francolin and Common Buzzard were not recorded in the dry season, and similarly, the Yellow-billed Kite and White-headed Vulture were not recorded in the wet season. During the dry season, four, 10 and 21 species were classified as common, frequent, and uncommon, respectively. During the wet season, one, 17 and 18 species were common, frequent, and uncommon, respectively. Rare and abundance species were not recorded under the two seasons (Table 4). No species were recorded as rare or abundant in either of the two seasons (Table 4).

**Table 4:** Encounter rate(n/point) of individual species in different abundance categories during both season/ number per Erica habitat effort.

Species	DRY			WET		
	ER		cv.ER	ER		cv.ER
Red-wing Starling	1.95	Common	0.56	0.79	Frequent	0.62
Ethiopian Siskin	1.64	Common	0.40	0.44	Frequent	0.26
Moorland Chat	1.38	Common	0.21	0.85	Frequent	0.30
Thekla lark	1.17	Common	0.24	0.65	Frequent	0.28
Ground-Scarper Thrush	0.73	Frequent	0.36	0.69	Frequent	0.37
Wattled Ibis	0.70	Frequent	0.37	0.81	Frequent	0.27
African Stonechat	0.57	Frequent	0.25	0.25	Frequent	0.28
Scaly Francolin	0.45	Frequent	0.25	0.36	Frequent	0.36
Streaky Seedeater	0.43	Frequent	0.21	0.37	Frequent	0.33
Mountain Thrush	0.43	Frequent	0.32	0.27	Frequent	0.29
Ethiopian Cistocola	0.40	Frequent	0.34	0.14	uncommon	0.43
Thick-billed Raven	0.35	Frequent	0.44	0.45	Frequent	0.35
Rouget’s Rail	0.27	Frequent	0.48	0.27	Frequent	0.45
Brown Rumped-seedeater	0.27	Frequent	0.35	0.21	Frequent	0.35
Brown Parisoma	0.19	Uncommon	0.48	0.17	uncommon	0.35
Common House Martin	0.18	Uncommon	1.00	0.29	Frequent	1.00
Moorland Francolin	0.17	Uncommon	0.87	0.10	uncommon	0.48
Cinnamon Bracken Warbler	0.14	Uncommon	0.55	0.02	uncommon	0.70
Abyssinian Slaty Flycatcher	0.14	Uncommon	0.74	0.12	uncommon	0.42
White-Collard Pigeon	0.12	Uncommon	0.71	0.19	uncommon	0.72
Pallid Harrier	0.12	Uncommon	0.49	0.08	uncommon	0.45
Cap Crow	0.10	Uncommon	0.70	0.24	Frequent	0.57
Tacazze Sunbird	0.07	Uncommon	0.74	0.35	Frequent	0.23
Lammergier	0.07	Uncommon	0.74	0.14	uncommon	0.45
African Snipe	0.07	Uncommon	1.00	0.07	uncommon	1.00
Hooded Vulture	0.06	Uncommon	0.71	0.04	uncommon	1.00
Mottled Swift	0.05	Uncommon	1.00	0.21	Frequent	1.00
Montane Nightjar	0.04	Uncommon	0.74	0.18	uncommon	0.49
Masachet Sunbird	0.04	Uncommon	1.00	0.10	uncommon	0.70
African Dusky flycatcher	0.04	Uncommon	0.74	0.07	uncommon	0.56
White Headed vulture	0.04	Uncommon	0.74	0	0	0
Scare Swift	0.02	Uncommon	0.70	1.02	Common	0.64
Rupplis Vulture	0.01	Uncommon	1.00	0.02	Uncommon	1.00
Yellow Billed Kite	0.01	Uncommon	1.00	0	0	0
Augur Buzzard	0.01	Uncommon	1.00	0.08	Uncommon	0.45
Chestnut-napped Francolin	0	0	0	0.07	Uncommon	1.00
Common Buzzard	0	0	0	0.01	Uncommon	1.00
African Citril	0.01	Uncommon	0.01	0		0
Sacred Ibis	0		0	0.02	Uncommon	0.02

**Notes.**

n total number of observations ER Encounter rate se.ER Standard error for ER cv.ER coefficient variation of ER

**Table 5:** Encounter rate(n/point) of individual species in different abundance categories during both season/number per natural forest habitat effort.

Species	Dry			Wet		
	ER		cv.ER	ER		cv.ER
Montane White-eye	1.88	Common	0.19	1.57	Common	0.21
Red-wing Starling	1.70	Common	0.37	0.88	Frequent	0.58
Black-winged Lovebird	0.49	Frequent	0.38	0.59	Frequent	0.35
Abyssinian Catbird	0.42	Frequent	0.27	0.34	Frequent	0.28
Streaky Seedeater	0.42	Frequent	0.29	0.30	Frequent	0.24
White-backed Black Tit	0.35	Frequent	0.28	0.27	Frequent	0.34
Brown Rumped-seedeater	0.35	Frequent	0.29	0.23	Frequent	0.32
Mountain Thrush	0.31	Frequent	0.28	0.26	Frequent	0.32
White-checked Turaco	0.30	Frequent	0.31	0.32	Frequent	0.28
Wattled Ibis	0.30	Frequent	0.40	0.53	Frequent	0.48
Thick-billed Raven	0.26	Frequent	0.35	0.40	Frequent	0.28
Mouse-colored Penduline-tit	0.25	Frequent	0.77	0	0	0
Baglafecht Weaver	0.24	Frequent	0.38	0.62	Frequent	0.30
Tacazze Sunbird	0.23	Frequent	0.30	0.20	Frequent	0.29
Yellow-bellied Waxbill	0.23	Frequent	0.48	0.17	uncommon	0.50
Common Bulbul	0.22	Frequent	0.45	0.21	Frequent	0.33
Cap Crow	0.19	Uncommon	0.51	0.22	Frequent	0.46
Ethiopian Siskin	0.19	Uncommon	0.53	0.19	uncommon	0.53
Red-collard Widow Bird	0.16	Uncommon	0.16	0.22	Frequent	0.77
Dusky turtle Dove	0.14	Uncommon	0.42	0.21	Frequent	0.34
Red-eyed Dove	0.13	Uncommon	0.36	0.09	uncommon	0.39
Eastern grey woodpecker	0.13	Uncommon	0.42	0.16	uncommon	0.40
Moorland Chat	0.11	Uncommon	0.77	0.06	uncommon	0.60
African Olive Pigeon	0.10	Uncommon	0.50	0.16	uncommon	0.37
Variable Sunbird	0.10	Uncommon	0.50	0	0	0
Chestnut-napped Francolin	0.09	Uncommon	1.00	0.23	Frequent	0.58
African Citril	0.09	Uncommon	0.46	0.20	Frequent	0.31
Yellow-fronted Parrot	0.09	Uncommon	0.74	0.14	uncommon	0.59
African Dusky flycatcher	0.09	Uncommon	0.52	0.13	uncommon	0.37
Tawny flanked Prina	0.09	Uncommon	0.38	0.07	uncommon	0.42
Abyssinian Slaty Flycatcher	0.09	Uncommon	0.38	0.03	uncommon	0.70
Speckled Mousebird	0.08	Uncommon	0.72	0.16	uncommon	0.61
Abyssinian Woodpecker	0.08	Uncommon	0.59	0.11	uncommon	0.46
White-Collard Pigeon	0.08	Uncommon	0.72	0.02	uncommon	0.74
Ethiopian Boubou	0.06	Uncommon	0.58	0.10	uncommon	0.43
Scaly Francolin	0.05	Uncommon	0.74	0.15	uncommon	0.50
African Paradise Flycatcher	0.05	Uncommon	0.50	0.06	uncommon	0.45
Cinnamon Bracken Warbler	0.05	Uncommon	0.46	0.06	uncommon	0.38
Yellow Crown Canary	0.05	Uncommon	0.74	0.20	Frequent	0.47
Abyssinian Forest Oriole	0.05	Uncommon	0.74	0.19	uncommon	0.50
Northern Puff back	0.05	Uncommon	0.74	0.06	uncommon	0.60
Augur Buzzard	0.04	Uncommon	0.66	0.05	uncommon	0.57
Yellow Wagtail	0.03	Uncommon	1.00	0.07	uncommon	0.71
Eurasian Hoopoe	0.03	Uncommon	0.70	0.06	uncommon	0.49
Abyssinian Ground Hornbill	0.03	Uncommon	1.00	0.05	uncommon	0.74
Narnia Trogon	0.03	Uncommon	0.60	0.05	uncommon	0.46
Africa Wooded Owl	0.03	Uncommon	0.60	0.02	uncommon	0.70
Mountain Wagtail	0.02	Uncommon	0.57	0.05	uncommon	0.57
Grey-backed Camaroptera	0.02	Uncommon	0.57	0.03	uncommon	0.49
Swanson’s Sparrow	0.02	Uncommon	1.00	0.02	uncommon	1.00
Ruppell’s Robin-Chat	0.02	Uncommon	1.00	0.04	uncommon	0.59
Tawny Eagle	0.02	Uncommon	0.70	0.02	uncommon	0.57
Hooded Vulture	0.02	Uncommon	1.00	0.02	uncommon	1.00
Abyssinian Owl	0.02	Uncommon	1.00	0	0	0
African Stonechat	0.02	Uncommon	1.00	0	0	0
Common Buzzard	0.01	Rare	1.00	0	0	0
Abyssinian Ground Thrush	0.01	Rare	0.53	0.04	uncommon	0.52
White Headed vulture	0	0	0	0.05	uncommon	0.52
Yellow Billed Kite	0	0	0	0.02	Uncommon	1.00

**Notes.**

n total number of observations ER Encounter rate se.ER Standard error for ER cv.ER coefficient variation of ER

**Table 6:** Encounter rate(n/point) of individual species in different abundance categories during both season/number per plantation forest habitat effort.

Species	Dry			Wet		
	ER		cv.ER	ER		cv.ER
Ground-Scarper Thrush	3.10	Common	0.67	0.20	Uncommon	0.47
Red-eyed Dove	2.80	Common	0.29	0.20	Uncommon	0.31
Montane White-eye	2.20	Common	0.62	0.20	Uncommon	0.61
Yellow Crown Canary	1.00	Frequent	0.63	2.00	Common	0.51
African Black Swift	1.00	Frequent	1.00	0	0	0
Baglafecht Weaver	0.80	Frequent	1.00	1.80	Common	0.67
African Citril	0.60	Frequent	1.00	0.60	Frequent	1.00
Tacazze Sunbird	0.50	Frequent	0.63	0.60	Frequent	0.67
Eurasian Hoopoe	0.40	Frequent	1.00	0.20	Uncommon	1.00
Speckled Mousebird	0.40	Frequent	1.00	0.20	Uncommon	0.73
Brown Rumped-seedeater	0.40	Frequent	1.00	0	0	0
Swanson’s Sparrow	0.40	Frequent	1.00	0	0	0
Semi-colored flycatcher	0.30	Frequent	1.50	0.20	Uncommon	1.00
Ethiopian Boubou	0.20	Uncommon	1.00	0.40	Frequent	0.73
African Dusky flycatcher	0.20	Uncommon	1.00	0.30	Frequent	1.00
Abyssinian Slaty Flycatcher	0.20	Uncommon	1.00	0.20	Uncommon	1.00
Dusky turtle Dove	0.20	Uncommon	1.00	0.20	Uncommon	1.00
Ethiopian Siskin	0.20	Uncommon	1.00	0	0	0
Streaky Seedeater	0.18	Uncommon	0.67	0.20	Uncommon	0.43
White Headed vulture	0.02	Uncommon	0.70	0	0	0
Common Bulbul	0	0	0	0.20	Uncommon	1.00
Mountain Thrush	0	0	0	0.20	Uncommon	1.00
Pied Crow	0	0	0	0.20	Uncommon	1.00
Thick-billed Raven	0	0	0	0.20	Uncommon	1.00
Augur Buzzard	0	0	0	0.10	Uncommon	1.00
Yellow Billed Kite	0	0	0	0.10	Uncommon	1.00

**Notes.**

n total number of observations ER Encounter rate se.ER Standard error for ER cv.ER coefficient variation of ER

In the dry afromontane forest habitat, the encounter rate was calculated as number of individual observation per the dry afromontane forest habitat effort (n/128). Montane White-eye was the highest relative abundance during both seasons (1.88 and 1.57). Mouse-colored Penduline Tit, Variable Sunbird, Abyssinian owl, African Stonechat and Common Buzzard were not recorded in the dry season, while in the wet season, Yellow-billed Kite and White-headed Vulture were not recorded. During the dry season two, 14, 39 and two species were common, frequent, uncommon, and rare respectively. During the wet season, one, 19 and 34 species were common, frequent and uncommon respectively. Rare and abundant species were not recorded under the wet season (Table 5). In the plantation forest habitat, the encounter rate was calculated as number of individual observations per plantation forest habitat Effort (n/10). Ground Scarper Thrush was the highest relative abundance during dry seasons (3.1). In the wet season, the yellow crown canary was the highest (2.00). During the dry season two, 10 and 7 species were common, Frequent and Uncommon, respectively. During the wet season, two, four and 14 species were common, Frequent and Uncommon respectively. Rare and abundance species were not recorded under two seasons. six species in the dry season and five species in the wet season were isolated record in the season (Table 6).

### Habitat association of bird species

**Figure 10:**
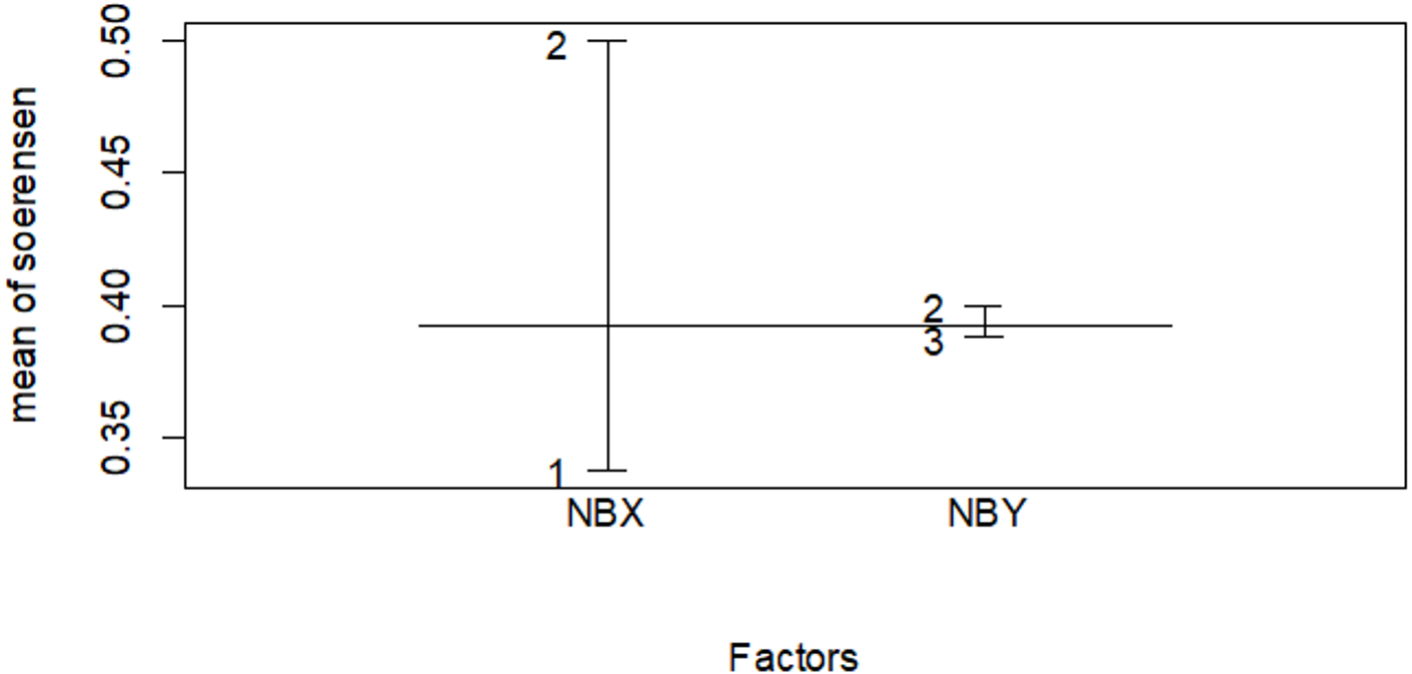
Similarity index among habitat types in the wet season. The numbers indicate 1 for the Erica habitat, 2 for the natural forest habitat and three for the plantation forest habitat, where NBX and NBY represents two comparable habitats .

**Figure 11:**
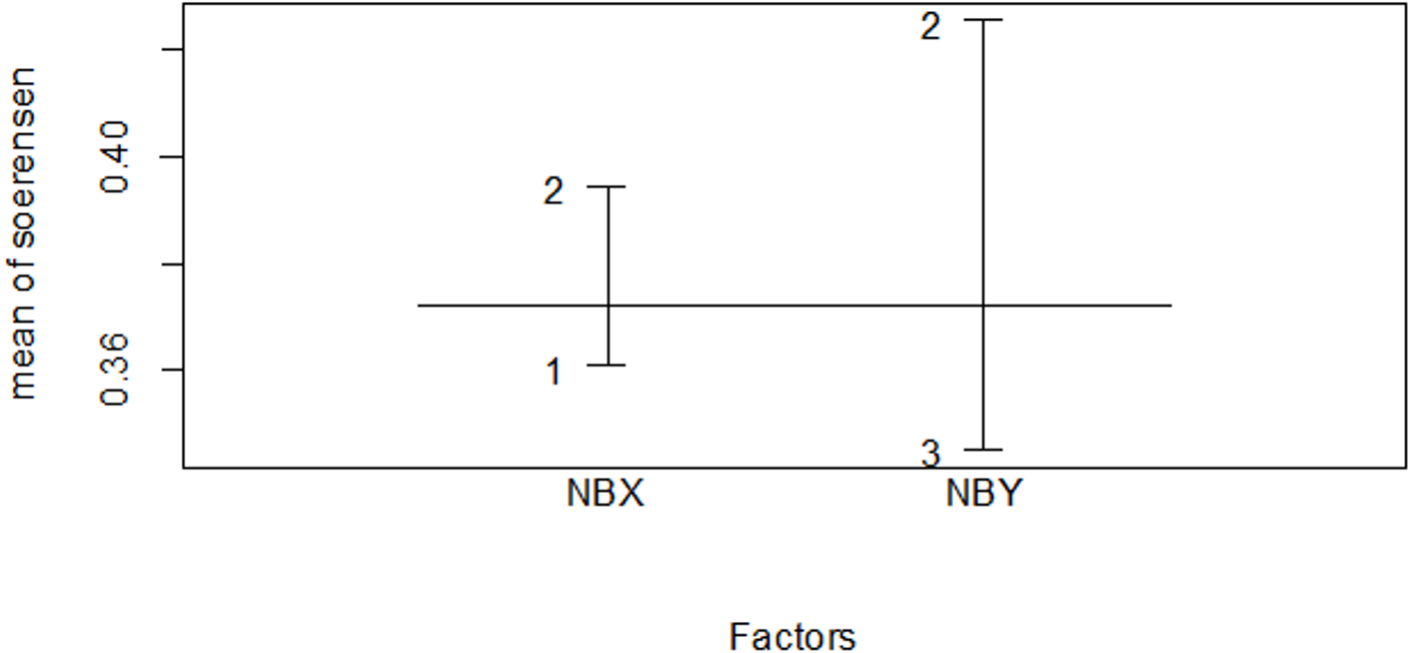
Similarity index among habitat types in the dry season. The numbers indicate 1 for the Erica habitat, 2 for the natural forest habitat and 3 for the plantation forest habitat, where NBX and NBY represents two comparable habitats.

Not all species were distributed in all habitat type. Of 39 different bird species, 15 species specific to Ericaceous sub-afro alpine scrubland vegetation (Table 4), while in Afromontane forest habitat, a number of 59 different bird species were founded, about twenty six species were specific to the habitat (Table 5), In the plantation forest habitat 26 bird species were recorded, there three species specific to community plantation forest habitat (Table 6) and the rest 34 species were recorded either in three or in only the two habitats (Appendix S1). A forest habitat accounts for a high number of bird species and high specific species. The Erica and forest habitats share more species that are common in the dry season (Fig. 10 and Table 7). Plantation and forest share more species that are common in the wet season (Fig. 11 and Table 7). Plantation and Erica share the lowest common species (Table 7).

## Discussion

### Low detection frequencies and detection probabilities

Some species, including the African Black Swift, Pied Crow, and Common Buzzard, exhibited low detection frequencies, falling below the standard recommended threshold of 60–80 observations. This could introduce bias into results, as insufficient data for these species may hinder robust analyses (Buckland et al., 1993). Additionally, certain species, particularly those specialized in woodland habitats, displayed lower detectability. This lower detectability, especially for species in closed habitats, may be influenced by various factors, including habitat structure and observer bias (Johnston et al., 2014). For multispecies surveys, it is crucial to account for local habitat effects on all species, not just those with abundant data (Zipkin et al., 2010).

### Seasonal variability

The research unveiled substantial seasonal fluctuations in bird abundance, with marked differences between the dry and wet seasons. While data collection was successful in both seasons, some species exhibited stronger presence during the wet season. This observation aligns with existing research emphasizing the influence of seasonal changes in resource availability and weather conditions on avian populations (French & Rockwell, 2011; Li et al., 2022). However, the effect of seasonality on avian species composition may be less pronounced in tropical regions. Seasonal changes in bird populations, feeding habits, and migration patterns are more prominent in temperate regions (Ward, 1969; White, Warren & Baines, 2015). Many birds in the study area are resident breeders, with limited migration during seasonal shifts, possibly contributing to the insignificant effect of seasons on bird species composition (Appendix S1). Migratory birds in Ethiopia are primarily associated with aquatic, wetland, and riverine habitats (Brooks, 2009).

**Table 7:** Species similarity index (SI) (Sorensen, 1948) among the three habitat types.

	Dry season	Wet season
Association	Sim	
Erica *vs* forest	0.425	0.400
Erica *vs* plantation	0.296	0.276
Planation *vs* forest	0.395	0.500

### Habitat influence on bird composition and structure

Habitat strongly influenced bird species composition, with distinct preferences observed among different species. Some species displayed specific habitat associations, such as the White-backed Black Tit in forest habitat, Thekla Lark in Erica, and Semi-colored Flycatcher in plantation habitat (Tables 4, 5 and 6). This suggests that avian community composition in the Ethiopian Highlands is intricately linked to habitat types. These findings align with previous studies highlighting the importance of habitat characteristics in shaping avian communities (Aynalem & Bekele, 2008). This suggests that various bird species have adapted to distinct ecological niches within the Ethiopian Highlands. The high species abundance in natural forest habitats further underscores their significance in avian biodiversity conservation. These findings resonate with studies by Hendershot et al. (2020), which underscored the importance of preserving diverse habitat types for effective avian biodiversity conservation. The heightened encounter rate observed within plantation forests (Table 6) suggests the presence of edge effects, particularly as influenced by adjacent agricultural areas. These edge effects are known to attract generalist bird species (Khamcha et al., 2018), which typically exploit transitional zones. However, it is noteworthy that the elevated encounter rate is primarily attributed to a select few bird species that have specifically adapted to the plantation forest habitat.

Microclimate and habitat structure emerged as major drivers influencing avian community composition within specific habitats (Rajpar & Zakaria, 2015). The relationship between habitat and species composition was further evident in the similarity index results, which indicated higher similarity between neighboring habitats (Table 5). Microclimate, habitat structure, and environmental gradients likely contribute to species distribution patterns and preferences within the Ethiopian Highlands.

Altitudinal gradients played a role in avian diversity, with the highest species composition recorded in middle elevation zones, primarily within forest habitats (Quintero & Jetz, 2018). Decreases in diversity at higher altitudes may be attributed to factors such as lower speciation or higher extinction rates, potentially influenced by smaller areas or lower temperatures (Quintero & Jetz, 2018).

The size of the habitat patch and edge effects may also influence avian species composition. Edge-sensitive, neutral, and preferring species respond differently to habitat edges (Brand & George, 2001). Forest species exhibit sensitivity to the contrast between natural and anthropogenic habitats (Zurita et al., 2012). Plantation habitats, characterized by smaller areas, displayed lower species composition estimates (Quintero & Jetz, 2018). As habitat destruction is a significant concern, particularly in forested areas, preserving diverse habitats and their associated bird species should be a conservation priority (Girma et al., 2017; Wang et al., 2017).

Future research in this region should address the limitations of our study. Long-term monitoring with extended survey periods, including intermediate seasons, can provide a more comprehensive understanding of avian population dynamics. In addition, expanding taxonomic coverage and accounting for external factors such as climate change and invasive species will enhance our understanding of avian biodiversity in the Ethiopian Highlands.

## Conclusion

The study revealed the presence of three unique endemic bird species, constituting a notable 20% of Ethiopia’s endemic avian population. Moreover, within the study area, an impressive 71% of Ethiopia and Eritrea’s endemic bird species call this region home, highlighting the exceptional levels of endemism present. These findings create an opportunity for the development of community-based ecotourism initiatives. Significantly, bird observation within various blocks of the study area is a crucial aspect. Rather than being influenced by seasonal fluctuations, these blocks are distinguished by differences in elevation, vegetation types, and the presence or absence of bird species. These variations in elevation generate microclimates, each nurturing distinct bird communities. However, this localized endemism also presents challenges, including the concentration of endemic species and potential resource constraints that could pose risks to specific bird populations. The study underscores the critical need for sustained surveillance and conservation strategies, particularly targeting forest-dependent and Erica-specific avian species. These proactive measures are imperative to address the potential risks associated with resource limitations and to safeguard the continued existence of these distinct bird communities. Our findings serve as a call to action for conservationists and policy makers, emphasizing the importance of preserving these unique ecosystems for future generations.

Thus, it is vital to prioritize dedicated conservation efforts, incorporating a multifaceted approach involving community-based ecotourism development and landscape restoration projects. This study represents the inaugural avian survey within this ecologically significant region. It establishes the groundwork for future research endeavors that can explore various aspects of this ecosystem. These future inquiries may investigate relationships between forest fragmentation and bird density, the impact of human disturbance on bird populations, and the intricate interplay between vegetation and bird communities. As this initial exploration concludes, it opens doors to a wealth of forthcoming insights and discoveries aimed at preserving the Dodola dry afromontane forest and ericaceous scrubland ecosystems.

## Supplemental Information

10.7717/peerj.16775/supp-1 Supplemental Information 1 The study data and analysis in distance software

10.7717/peerj.16775/supp-2 Supplemental Information 2 Species accumulation r source code

10.7717/peerj.16775/supp-3 Supplemental Information 3 Codes for each species discovered during sampling and number of these species’ observation

10.7717/peerj.16775/supp-4 Supplemental Information 4 Checklist of The Birds of Dodola Dry Evergreen Afromontane Forest and Sub-Afro Alpine Scrubland Vegetation

## Additional Information and Declarations

### Data Availability

The following information was supplied regarding data availability: The code is available in the Supplementary File.
