# Supplementary material for: Species composition, relative abundance, and habitat association of birds in Dodola dry evergreen afro-montane forest and sub-afro-alpine scrubland vegetation, southeast Ethiopia
Source: PeerJ. 2024 Jan 11;12:e16775. doi: 10.7717/peerj.16775 (PMC10788088; doi:10.7717/peerj.16775)
Supplement: Supplemental Information 4 [file peerj-12-16775-s004.docx]

## **Appendix 1** **Checklist of The Birds of Dodola Dry Evergreen Afromontane Forest and Sub-Afro Alpine Scrubland Vegetation**

Number of species: 78

Number of endemics: 3

Endemics Ethiopia and Eritrea: 10

Number of globally threatened species: 6

Number of near threatened species: 3

Number of Resident breading:67, rb:3

Number of NB: 4; nb: 2

MB/NB:1

Uncertain record(?

Table 8 **Checklist of The Birds of Dodola Dry Evergreen Afromontane Forest and Sub-Afro Alpine Scrubland Vegetation**

| Common Name | Scientific name | Family | Order | IUCN | status |
| --- | --- | --- | --- | --- | --- |
| Wattled Ibis | *Bostrychia carunculate* | Threskiornithidae | PELECANIFORMES |  | RB |
| Sacred Ibis | *Threskiornis aethiopicus* | Threskiornithidae | PELECANIFORMES |  | RB |
| Yellow-billed Kite | *Milvus aegyptius* | Accipitridae | ACCIPITRIFORMES | NR | RB |
| Lammergeier | *Gypaetus barbatus* | Accipitridae | ACCIPITRIFORMES | NT | RB |
| Hooded Vulture | *Necrosyrtes monachus* | Accipitridae | ACCIPITRIFORMES | CR | RB |
| Ruppll's Vulture | *Gyps rueppellii* | Accipitridae | ACCIPITRIFORMES | CR | RB |
| White-headed Vulture | *Trigonoceps occipitalis* | Accipitridae | ACCIPITRIFORMES | CR | Rb |
| Pallid Harrier | *Circus macrourus* | Accipitridae | ACCIPITRIFORMES | NT | NB |
| Common Buzzard | *Buteo buteo* | Accipitridae | ACCIPITRIFORMES |  | NB |
| Augur Buzzard | *Buteo augur* | Accipitridae | ACCIPITRIFORMES |  | RB |
| Tawny Eagle | *Aquila rapax* | Accipitridae | ACCIPITRIFORMES | VU | RB |
| Moorland Francolin | *Scleroptila psilolaemus* | Phasianidae | GALLIFORMES |  | RB |
| Scally Francolin | *Pternistis squamatus* | Phasianidae | GALLIFORMES |  | RB |
| Chestnut-napped Francolin | *Pternistis castaneicollis* | Phasianidae | GALLIFORMES |  | RB |
| Rouget's Rail | *Rouget`s rougetii* | Rallidae | GRUIFORMES | NT | RB |
| African Snipe | *Gallinago gallinago* | Scolopacidae | CHARADRIIFORMES |  | RB |
| African Olive Pigeon | *Columba arquatrix* | Columbidae | COLUMBIFORMES: |  | RB |
| White-collard Pigeon | *Columba albitorques* | Columbidae | COLUMBIFORMES: |  | RB |
| Red-eyed Dove | *Streptopelia roseogrisea* | Columbidae | COLUMBIFORMES: |  | RB |
| Dusky Turtle Dove | *Streptopelia lugens* | Columbidae | COLUMBIFORMES: |  | RB |
| Yellow-fronted Parrot | *Poicephalus flavifrons* | Psittacidae | PSITTACIFORMES |  | RB |
| Black-winged Lovebird | *Agapornis taranta* | Psittacidae | PSITTACIFORMES |  | RB |
| White-cheeked Turaco | *Tauraco leucotis* | Musophagidae | MUSOPHAGIFORMES |  | RB |
| Abyssinian Owl | *Asio abyssinicus* | Strigidae | STRIGIFORMES |  | RB |
| African Wooded Owl | *Strix woodfordii* | Strigidae | STRIGIFORMES |  | RB |
| Montane Nightjar | *Caprimulgus poliocephalus* | Caprimulgidae | CAPRIMULGIFORMES |  | RB |
| Scarce Swift | *Schoutedenapus myoptilus* | Apodidae | CAPRIMULGIFORMES |  | Nb |
| African Black Swift | *Cypseloides niger* | Apodidae | CAPRIMULGIFORMES | VU | ? |
| Mottled Swift | *Tachymarptis aequatorialis* | Apodidae | CAPRIMULGIFORMES |  | Rb |
| Speckled Mousebird | *Colius striatus* | Coliidae | COLIIFORMES |  | RB |
| Narina Trogon | *Apaloderma narina* | Trogonidae | TROGONIFORMES |  | RB |
| Eurasian Hoopoe | *Upupa epops* | Upupidae | BUCEROTIFORMES |  | MB/NB |
| Abyssinian Ground-hornbill | *Bucorvus abyssinicus* | Bucorvidae | BUCEROTIFORMES | VU | RB |
| Abyssinian Woodpecker | *Dendropicos abyssinicus* | Picidae | *PICIFORMES* |  | RB |
| Eastern Grey Woodpecker | *Dendropicos goertae* | Picidae | *PICIFORMES* |  | RB |
| Thekla Lark | *Galerida theklae* | Alaudidae | PASSERIFORMES |  | RB |
| Common House Martin | *Delichon urbicum* | Hirundinidae | PASSERIFORMES |  | NB |
| Yellow Wagtail | *Motacilla flava* | Motacillidae | PASSERIFORMES |  | NB |
| Mountain Wagtail | *Motacilla clara* | Motacillidae | PASSERIFORMES |  | RB |
| Common Bulbul | *Pycnonotus barbatus* | Pycnonotidae | PASSERIFORMES |  | RB |
| Ruppll's Robin-chat | *Cossypha semirufa* | Turdidae | PASSERIFORMES |  | RB |
| African Stonchat | *Saxicola(torquatus) toquatus* | Turdidae | PASSERIFORMES |  | RB |
| Moorland Chat | *Cercomela sordida* | Turdidae | PASSERIFORMES |  | RB |
| Abyssinian Ground Thrush | *Zoothera piaggiae* | Turdidae | PASSERIFORMES |  | RB |
| Groundscarper Thrush | *Psophocichla litsitsirupa* | Turdidae | PASSERIFORMES |  | RB |
| Mountain Thrush | *Turdus(olivaceus) abyssinicus* | Turdidae | PASSERIFORMES |  | RB |
| Cinnamon Bracken Warbler | *Bradypterus alfredi* | Turdidae | PASSERIFORMES |  | RB |
| Brown Parisoma | *Parisoma lugens* | Turdidae | PASSERIFORMES |  | RB |
| Ethiopian Cisticola | *Cisticola(galactotes) lugubris* | Turdidae | PASSERIFORMES |  | RB |
| Tawny-flanked Prina | *Prinia subflava* | Turdidae | PASSERIFORMES |  | RB |
| Grey-backed Camaroptera | *Camaroptera superciliaris* | Turdidae | PASSERIFORMES |  | RB |
| Abyssinian Slaty Flycatcher | *Melaenornis chocolatinus* | Muscicapidae | PASSERIFORMES |  | RB |
| African Dusky Flycatcher | *Muscicapa adusta* | Muscicapidae | PASSERIFORMES |  | RB |
| Semi collard Flycatcher | *Ficedula semitorquata* | Muscicapidae | PASSERIFORMES |  | Nb |
| Abyssinian Catbird | *Parophasma galinieri* | Timaliidae | PASSERIFORMES |  | RB |
| African Paradise Flycatcher | *Terpsiphone viridis* | Monarchidae | PASSERIFORMES |  | RB |
| White-backed Black Tit | *Parus leuconotus* | Paridae | PASSERIFORMES |  | RB |
| Mouse-colored Penduline-tit | *Anthoscopus musculus* | Remizidae | PASSERIFORMES |  | RB |
| Tacazze Sunbird | *Nectarinia tacazze* | Nectariniidae | PASSERIFORMES |  | RB |
| Malachite Sunbird | *Nectarinia famosa* | Nectariniidae | PASSERIFORMES |  | RB |
| Variable Sunbird | *Cinnyris venustus* | Nectariniidae | PASSERIFORMES |  | RB |
| Montane White-eye | *Zosterops poliogastrus* | Zosteropidae | PASSERIFORMES |  | RB |
| Northen Puffback | *Dryoscopus gambensis* | Malaconotidae | PASSERIFORMES |  | RB |
| Ethiopian Boubou | *Laniarius aethiopicus* | Malaconotidae | PASSERIFORMES |  | RB |
| Abyssinian Oriole | *Oriolus monacha* | Oriolidae | PASSERIFORMES |  | RB |
| Cape Crow | *Corvus capensis* | Corvidae | PASSERIFORMES |  | RB |
| Pied Crow | *Corvus albus* | Corvidae | PASSERIFORMES |  | RB |
| Thick-billed Raven | *Corvus crassirostris* | Corvidae | PASSERIFORMES |  | RB |
| Red-wing Starling | *Onychognathus morio* | Sturnidae | PASSERIFORMES |  | RB |
| Swainson's Sparrow | *Passer swainsonii* | Passeridae | PASSERIFORMES |  | RB |
| Baglafecht Weaver | *Ploceus baglafecht* | Ploceidae | PASSERIFORMES |  | RB |
| Red-collard Widowbird | *Euplectes laticauda* | Ploceidae | PASSERIFORMES |  | RB |
| Yellow-bellied Waxbill | *Coccopygia quartinia* | Estrildidae | PASSERIFORMES |  | RB |
| African Citril | *Serinus hypostictus* | Fringillidae | PASSERIFORMES |  | RB |
| Streaky Seedeater | *Serinus striolatus* | Fringillidae | PASSERIFORMES |  | RB |
| Brown -rumped Seedeater | *Serinus trstriatus* | Fringillidae | PASSERIFORMES |  | RB |
| Yellow Crown Canary | *Serinus flavivertex* | Fringillidae | PASSERIFORMES |  | RB |
| Ethiopian Siskin | *Serinus nigriceps* | Fringillidae | PASSERIFORMES |  | RB |

***Key:***

**RB**=Resident Breeder**; rb**=mainly resident but partially erratic or migratory with range

**MB**= breading visitor (intra-African migrant), **mb**=breading visitor (sparse occurrence)

**NB**=non breading visitor (Palearctic or intra-African migrant), **nb**= non-breading visitor (sparse occurrence)

**v**= vagrant; **x** = isolated record; **?**= status uncertain; *= few record status
